# Impact of morphological traits and irrigation levels on fresh herbage yield of sorghum x sudangrass hybrid: Modelling data mining techniques

**DOI:** 10.1371/journal.pone.0318230

**Published:** 2025-02-05

**Authors:** Halit Tutar, Senol Celik, Hasan Er, Erdal Gönülal

**Affiliations:** 1 Faculty of Agriculture, Department of Field Crops, Bingöl University, Bingöl, Türkiye; 2 Faculty of Agriculture, Department of Animal Science, Bingöl University, Bingöl, Türkiye; 3 Faculty of Agriculture, Department of Biosystems Engineering, Bingöl University, Bingöl, Türkiye; 4 Bahri Dagdas International Agriculture Research Institute, Konya, Türkiye; Universitas Sebelas Maret, INDONESIA

## Abstract

In this study, the effect of morphological traits on fresh herbage yield of sorghum x sudangrass hybrid plant grown in Konya province, which is the largest cereal production area in Turkey, was analyzed with some data mining methods. For this purpose, Artificial Neural Networks (ANN), Automatic Linear Model (ALM), Random Forest (RF) Algorithm and Multivariate Adaptive Regression Spline (MARS) Algorithm were used, and the prediction performances of these methods were compared. Plant height of 251.22 cm, stem diameter of 7.03 mm, fresh herbage yield of 8010.69 kg da^-1^, crude protein ratio of 9.09%, acid detergent fiber 33.23%, neutral detergent fiber 57.44%, acid detergent lignin 7.43%, dry matter digestibility of 63.01%, dry matter intake 2.11%, and relative feed value of 103.02 were the descriptive statistical values that were computed. Model fit statistics, including coefficient of determination (R^2^), adjusted R^2^, root of mean square error (RMSE), mean absolute percentage error (MAPE), standard deviation ratio (SD ratio), Mean Absolution Error (MAE) and Relative Absolution Error (RAE), were used to evaluate the prediction abilities of the fitted models. The MARS method was shown to be the best model for describing fresh herbage yield, with the lowest values of RMSE, MAPE, SD ratio, MAE and RAE (137.7, 1.488, 0.072, 109.718 and 0.017, respectively), as well as the highest R^2^ value (0.995) and adjusted R^2^ value (0.991). The experimental results show that the MARS algorithm is the most suitable model for predicting fresh herbage yield in sorghum x sudangrass hybrid, providing a good alternative to other data mining algorithms.

## Introduction

Due to global climate change, temperature and atmospheric CO_2_ levels are rising, and droughts are becoming widespread [1]. Drought is the primary environmental stressor that restricts crop production in arid and semi-arid regions [2]. Severe drought can significantly reduce crop yield and quality, leading to scarcity [3]. Therefore, more drought-resistant plant varieties and water-saving cultivation systems should be used to better cope with changing climate conditions [4]. In drought-prone environments, sweet sorghum and sorghum x sudangrass (SSG) hybrids can be considered as alternatives to maize and wheat [5]. Sorghum (*Sorghum bicolor* L. Moench), as a forage crop, is a highly drought-resistant member of the grain family when compared to other crops in this family [6,7].

Sorghum is the fifth most important cereal crop worldwide, serving as a staple food for over 500 million people across more than 30 countries. Globally, it ranks as the fourth most significant grain [8,9]. Sorghum is a rich source of protein and fiber [10], and beyond its nutritional role, it is also utilized as a raw material for bioethanol production [11]. Despite its reputation for resilience, drought stress remains a major challenge, adversely affecting both productivity and nutritional quality in key production areas. Thus, understanding how drought impacts sorghum and how the plant responds is critical for improving its resistance to such stress [12].

Statistical models are widely used in forecasting studies due to their simplicity and low computational cost requirements [13,14]. Data mining algorithms offer a powerful alternative to traditional regression methods, addressing some limitations [15]. These methods have gained widespread popularity in Lately, for predicting plant yield and various traits, as well as classifying plants by species [16,17]. Data mining involves extracting valuable and meaningful information from large datasets and is a relatively new interdisciplinary field that focuses on data analysis and knowledge discovery [18,19]. This approach combines multiple techniques, such as statistical analysis, data visualization, neural networks, knowledge discovery, pattern recognition, and database management [20].

Data mining is an approach that offers practical and effective solutions for productivity and sustainability in the agricultural sector. Data mining techniques used for agricultural yield prediction provide farmers with the opportunity to make more accurate and timely decisions by analyzing multiple factors such as climate changes, soil properties, and plant growth dynamics [21]. These techniques contribute to the efficient use of agricultural inputs by optimizing production processes. In addition, forecasts obtained through data mining can also guide agricultural policy making and help to balance agricultural supply and demand. Thus, data mining contributes to both economic and environmental sustainability by strengthening decision support systems in the agricultural sector [22].

Various studies have been conducted on field crops and other plants using data mining methods. Numerous studies have demonstrated the potential of data mining algorithms for predicting agricultural production systems [23–25]. In studies by [26,27], the relationship between certain climate parameters and maize yield was examined. [28] aimed to evaluate the effectiveness of textural features in distinguishing seeds produced under normal or drought conditions using discriminant models. [29] researched biomass yield prediction for sorghum plants using remote and proximal sensing-based model algorithms.

In this study, the factors affecting fresh herbage yield of sorghum x sudangrass (SSG) hybrid were identified using data mining algorithms based on various plant traits. A comparative study was conducted by evaluating and comparing the performances of different algorithms such as Artificial Neural Network (ANN), Multi-Layer Perceptron Artificial Neural Networks (MLP), Random Forest (RF) algorithm, Multivariate Adaptive Regression Spline (MARS), and Automatic Linear Modeling (ALM).

## 2. Material and methods

### 2.1. Research area and datasets

**Fig 1:**
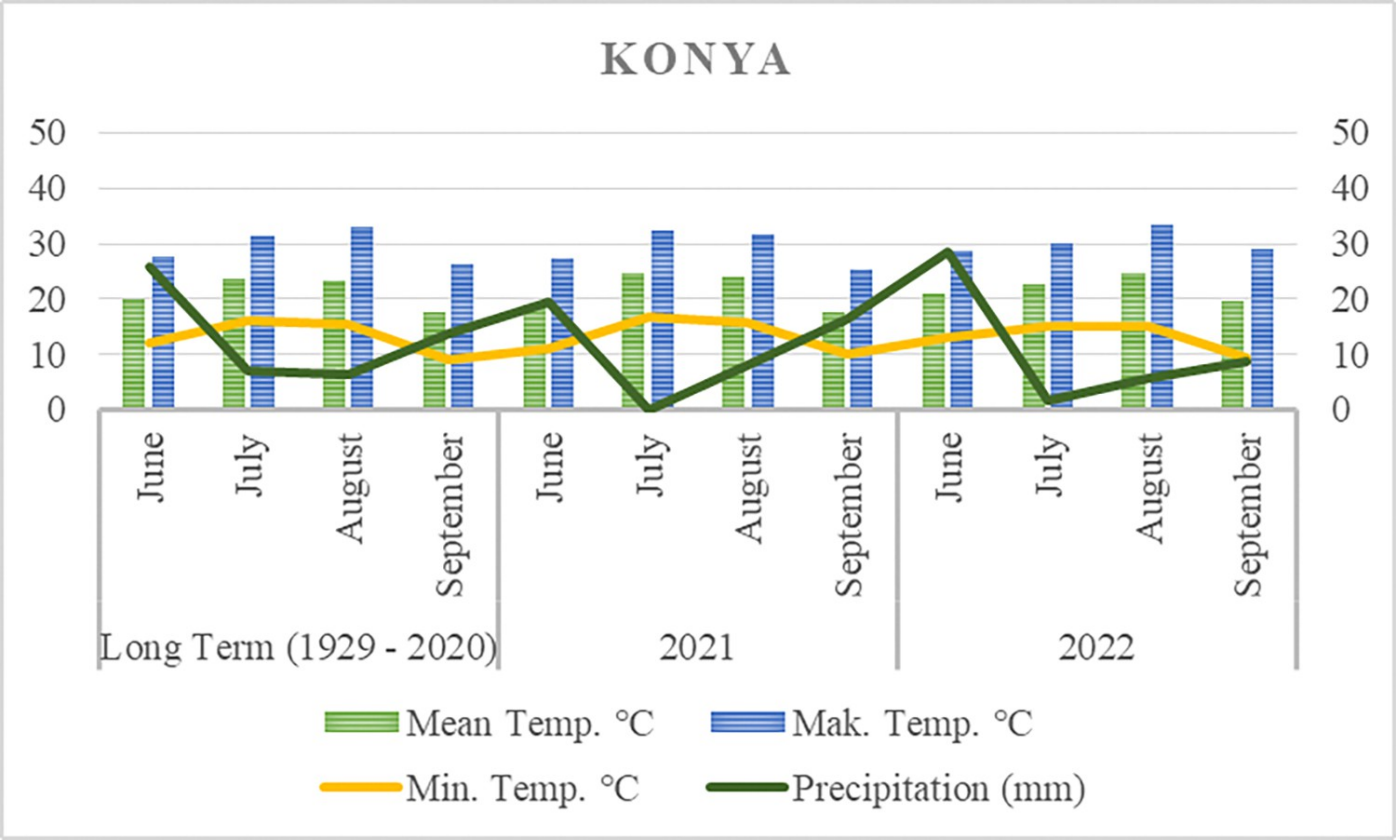
Average meteorological parameters during the plant’s growing period.

This research was conducted during the 2021 and 2022 growing seasons in Konya province, Türkiye, located between 37° 51´ N and 32° 33´ E. Konya is situated at an elevation of approximately 1006 meters above sea level. The study area has a semi-arid climate, with most of the annual rainfall occurring in the winter months. The recorded rainfall during the vegetation period of the SSG hybrid plant was determined to be 44 and 44.4 mm, respectively. The min., max., and average temperatures recorded in Konya during the 2021 and 2022 growing seasons align with the long-term average temperatures (Fig 1).

The dataset for the study was collected from the field between 2021 and 2022. The experimental dataset consists of parameters obtained under different irrigation levels (100% (I_100_), 75% (I_75_), 50% (I_50_), and 25% (I^25^) replenishment of water depleted from field capacity). The research includes 32 dataset parameters: Plant height (PH), stem diameter (SD), crude protein ratio (CPR), acid detergent fiber (ADF), neutral detergent fiber (NDF), acid detergent lignin (ADL), dry matter digestibility (DMD), dry matter intake (DMI), and relative feed value (RFV) as input parameters. Fresh herbage yield is the output parameter.

Soil analysis revealed that the study area had a clay-loam texture, free of salt, calcareous, and low in organic matter content (Table 1).

**Table 1:** Results of soil analysis from experimental fields.

Location	Depth (cm)	Texture	pH	EC (dSm^-1^)	CaCO_3_ (%)	Organic matter (%)	P_2_O_5_ (kg da^-1^)	K_2_O (kg da^-1^)
Konya	0–30	Clay-Loam	7.80	0.77	33.40	1.70	13.50	97.00

### 2.2. Experimental details

The field experiment followed a randomized block design with four replications. Each plot consisted of four rows, with a row spacing of 45 cm and a row length of 5 meters. Prior to sowing, 10 kg da^-1^ of NPK compound fertilizer was applied, and an additional 22 kg da^-1^ of nitrogen fertilizer was administered when the plants reached a height of 40–50 cm. The treatments were based on four different irrigation levels, where 100% (I_100_), 75% (I_75_), 50% (I_50_) and 25% (I_25_) of the water consumed from the field capacity was supplemented at 100% (I_100_), 75% (I_75_), 50% (I_50_) and 25% (I_25_), respectively, to fill the moisture deficit in the 0–90 cm soil layer, which was determined as the effective root depth of SSG hybrid. In 2021, the irrigation water applied for I_100_, I_75_, I_50_, and I_25_ was 510 mm, 395 mm, 280 mm, and 165 mm, respectively, while in 2022, these values were 480 mm, 370 mm, 260 mm, and 150 mm, respectively. Sowing took place in the first week of June, with harvesting completed by the last week of September. After mowing the plants in each plot, the fresh herbage yield was calculated on a per decare basis. For detailed analysis, ten plants were randomly selected from the harvested samples. The crude protein (CP), acid detergent fiber (ADF), and neutral detergent fiber (NDF) ratios were measured using a NIRS device. Additionally, dry matter digestibility (DMD), dry matter intake (DMI), and relative feed value (RFV) were calculated based on established formulas [30,31].


DMD=88.9−(0.779*%ADF) (1)

DMI=120/(%NDF) (2)

RFV=(DDM*DMI)/1.29 (3)

### 2.3. Data mining

Tree-structured classification and regression are alternative ways to classification and regression that do not rely on normalcy assumptions or user-specified model statements, as do certain earlier methods like discriminant analysis and ordinary least squares regression [32]. The data taken from various domains inherently consists of extremely correlated observations, which coincides with the exponential increase in the magnitude of the data that must be evaluated due to technology improvements. This phenomenon, known as multicollinearity, affects the performance of both statistics and machine learning methods. Statistical models presented as a potential solution to this problem have not received enough evaluation in the literature. As a result, tackling the multicollinearity problem requires a thorough comparison of statistical and machine learning models [33]. The literature proposes a variety of strategies for dealing with multicollinearity. Although the first recommended solution is to collect additional data, this may not always be feasible owing to cost constraints or even impossible. The second option is to use non-least squares techniques (such as ridge, liu, lasso, and elastic net regression). The third step is to modify the model by adding new or groups of variables based on the multi-correlated variables [34]. Lastly, as more popular solutions for multicollinearity or other issues (such as outliers) in the field of machine learning, a variety of preprocessing techniques are used, including as centering, scaling, normalization, and standardization [33]. Three strategies were put out by [35] to address the multicollinearity issue in the data mining domain: employ ridge regression, train a two-layer neural network with the instructor, and then successively modify a single-layer neural network.

Alternative models (such as the Random Forest algorithm, Multivariate Adaptive Regression Spline, Artificial Neural Network, and Automatic Linear Modeling) will be the main focus of this investigation.

#### 2.3.1. Artificial Neural Network (ANN)

The artificial neural network (ANN) is modeled to replicate the functioning of the human brain and nervous system. These neural network models are mathematical systems, inspired by biological neural networks, designed to mimic the processes of animal brains [36].

#### 2.3.2. Multi-Layer Perceptron Artificial Neural Networks (MLP)

The Artificial Neural Network (ANN) used in this study is a feed-forward Multi-Layer Perceptron (MLP) model, comprising an input layer, a single hidden layer, and an output layer. In this structure, the neurons in the input layer pass signals to the hidden layer, where the neurons are interconnected through weighted connections. Both the hidden and output layers use the same activation function. The MLP was trained for 1000 epochs.

The activation function is crucial because it controls the level of activation and the strength of the output signal of an artificial neuron. Non-linear activation functions are often used as they enable better performance in function approximation [37].

The hyperbolic tangent activation function can be expressed as follows [38]:

$$f(x)=\frac{1-e^{-ax}}{1+e^{-ax}}$$ (4)

#### 2.3.3. Random Forest (RF) algorithm

For both regression and classification tasks, the Random Forest (RF) algorithm is a powerful ensemble learning technique [39]. This method addresses the overfitting problem inherent in decision trees [40] by combining Breiman’s Bagging algorithm [41] with decision trees. RF demonstrates strong generalization capabilities and robustness to unseen data, while also performing exceptionally well on training data. By randomly selecting features from the candidate feature set, RF can handle large-scale, high-dimensional datasets and reduces the impact of feature correlations on the model. Additionally, RF enhances model stability by averaging the results of multiple decision trees, thereby mitigating the influence of noise [42].

The creation of a decision tree typically involves three steps: feature selection, decision tree generation, and pruning. The classification error rate is often estimated using the information gain, information gain ratio, or Gini index, which are commonly used as feature selection criteria. In addition to synthesizing multiple input features to enhance the model, the random forest approach can also assess the relative importance of input features. By combining the decision outcomes of several decision trees, the random forest method, as an ensemble learning algorithm, can filter out anomalous data, paving the way for input feature optimization and improved model accuracy. Decision trees, the foundational components of random forests, possess strong generalization capabilities that allow them to address regression problems as well as classification tasks [43].

Furthermore, RF is exceptionally fast, highly resistant to overfitting, and allows users to generate as many trees as desired [44].

The expected values for unknown instances x are calculated by averaging the predictions from all the regression trees, as shown below:

$$f(x)=\frac{1}{B}\sum_{b=1}^{B}t_{b}(x)$$ (5)

Bagging involves repeatedly drawing *B* bootstrap samples from the training data and fits *t*_*b*_ trees based on the Gini impurity for each sample.

In this study, a Random Forest (RF) model was trained using an ensemble of 500 regression trees, with all available factors as predictors and total tree height as the response variable [45]. The RF model was implemented with the R package "ranger," utilizing default hyperparameter settings. The "ranger" package was selected for its faster data analysis capabilities and more efficient memory usage compared to other widely used random forest packages in R [46].

#### 2.3.4. Multivariate adaptive regression spline (MARS)

In 1991, Friedman introduced the multivariate adaptive regression spline (MARS) technique [47,48]. Typically, a regression pair is represented as (X_i_, Y_i_), where X_i_ corresponds to one or more independent variables and Y_i_ is the dependent variable. In the MARS model, each independent variable is associated with one or more split points (t_i_). For X_i_ ≥ t_i_, the resulting equation is known as the right-side basis function (BF), while for X_i_ < t_i_, the equation is called the left-side basis function. These left and right basis functions (spline functions) establish the relationship between X_i_ and the dependent variable Y_i_. The following equations present the mathematical expressions for the right and left basis functions [49].

$${\left[-(X_{i}-t_{i})\right]}_{+}^{q}=\left\{ \begin{array}{c} {\left(t_{i}-X_{i}\right)}^{q}\;If\;X_{i}<t_{i}\\ 0\;otherwise \end{array}\right.$$ (6)

$${\left[+(X_{i}-t_{i})\right]}_{+}^{q}=\left\{ \begin{array}{c} {\left(X_{i}-t_{i}\right)}^{q}\;If\;X_{i}\geq t_{i}\\ 0\;otherwise \end{array}\right.$$ (7)

where q (≥0) is the power to which the splines are raised and which defines the degree of smoothness of the outcome function estimate.

The MARS model may be expressed as follows: [50].


$$f(x)=\sum_{m=1}^{m}a_{m}B_{m}(x)$$ (8)

In this context,f (x) denotes the MARS model, and *B*_*m*_(*x*) represents the basis function. The index of the basis function is indicated by *m*, while the total number of basic functions in the MARS model is also represented by *m*. The coefficient corresponding to the m-th basis function is labeled as a_m_, and x ∈ R^n^ signifies the vector of predictor variables. The MARS model constructs the basis functions in a product form.


$$B_{m}(x)=\prod_{k=1}^{k_{m}}b_{km}$$ (9)

In this context, *b*_*km*_ represents the k-th univariate function within *B*_*m*_(*x*), and *k*_*m*_ indicates the total number of univariate terms multiplied in *B*_*m*_(*x*). When *k*_*m*_>1, *k*_*m*_ refers to the degree of the interaction term. On the other hand, if *k*_*m*_ = 1, the basis function is univariate. Each basis function contains refraction points, which serve as the knots for that function. The simplest form of *b*_*km*_ consists of truncated linear functions, structured as follows:

$$b(x/t)={\left[+(x-t)\right]}_{+}=max\{+(x-t),0\}$$ (10)

or

$$b(x/t)={\left[-(x-t)\right]}_{+}=max\{-(x-t),0\}$$ (11)

where the location t is called the knot of the basis function.

The MARS approach selects models using the GCV criterion [51]. The GCV criteria is used to assess the degree of fit and accuracy of the model [52]. GCV coefficient [53,54], as shown in the equation below.

$$GCV=\frac{\sum_{i=1}^{n}{\left(y_{i}-\hat{y_{i}})\right)}^{2}}{{\left[1-\frac{M(\lambda )}{n}\right]}^{2}}$$ (12)

where n is the quantity of sample data, C is the cost-complexity measure of the new basic functions, and *M*(*λ*) is the number of regression models created by the MARS model [55].

#### 2.3.5. Automatic Linear Modeling (ALM)

Automatic Linear Modeling (ALM) enables researchers to automatically select the optimal subset of predictors. To enhance data fit, ALM directly transforms predictors. When conducting ALM analysis, SPSS utilizes techniques such as time rescaling, outlier reduction, category merging, and other methods. While ALM can be applied to small and medium-sized datasets, it is particularly advantageous for large and complex datasets. The benefits of ALM become more evident, especially when dealing with multiple estimators [56–58]. Additionally, the ALM technique is highly effective for selecting and categorizing variables.

$$Y_{i}=\beta_{0}+\beta_{1}X_{i1}+\beta_{2}X_{i2}+\cdots +\beta_{n}X_{in}+\varepsilon$$ (13)

Where Yi is dependent or outcome variable, X_i_’s predictor or independent variables, β_0_ is constant, β_n_ is the slope coefficients for each predictor and *ε* is the error term [59,60].

The goodness of fit statistics are summarized as follows: [61].

**R^2^ (Coefficient of Determination):** Evaluates how well the model can predict future data and explain the variance within the observed dataset, playing a key role in verifying prediction accuracy.

**RMSE (Root Mean Squared Error):** Represents the average size of prediction errors, offering insight into the overall error magnitude.

**MAE (Mean Absolute Error):** Reflects the average of the absolute differences between predicted and actual values, making it easier to interpret error without considering its direction.

Both RMSE and MAE provide important measures of prediction error size, essential for evaluating forecast precision.

**RAE (Relative Absolute Error):** Provides a normalized error metric by comparing performance against a simple baseline model, making it useful for comparing different models.

Below are the formulas for these goodness of fit statistics [62–64].

Coefficient of Determination:

$$R^{2}=1-\frac{\sum_{i=1}^{n}{\left(y_{i}-\hat{y_{i}}\right)}^{2}}{\sum_{i=1}^{n}{\left(y_{i}-{\bar{y}}_{i}\right)}^{2}}$$ (14)

Adjusted Coefficient of Determination:

$${Adj.R}^{2}=1-\frac{\frac{1}{n-k-1}\sum_{i=1}^{n}{\left(y_{i}-\hat{y_{i}}\right)}^{2}}{\frac{1}{n-1}\sum_{i=1}^{n}{\left(y_{i}-{\bar{y}}_{i}\right)}^{2}}$$ (15)

Root-mean-square error (RMSE) given by the following formula:

$$RMSE=\sqrt{\frac{\sum_{i=1}^{n}{\left(y_{i}-\hat{y_{i}}\right)}^{2}}{n}}$$ (16)

Mean Absolute Error

$$MAE=\frac{\sum_{i=1}^{n}\left|y_{i}-\hat{y_{i}}\right|}{n}$$ (17)

Relative Absolute Error

$$RAE=\frac{\sum_{i=1}^{n}\left|y_{i}-\hat{y_{i}}\right|}{\sum_{i=1}^{n}\left|y_{i}-\bar{y_{i}}\right|}$$ (18)

In the methods, the following other model evaluation criteria were also calculated [65–68].

Mean absolute percentage error (MAPE);

$$MAPE=\frac{1}{n}\sum_{i=1}^{n}|\frac{Y_{i}-{\hat{Y}}_{i}}{Y_{i}}|.100$$ (19)

Standard Deviation Ratio;

$${SD}_{ratio}=\sqrt{\frac{\frac{1}{n-1}\sum_{i=1}^{n}{\left(\varepsilon_{i}-\bar{\varepsilon }\right)}^{2}}{\frac{1}{n-1}\sum_{i=1}^{n}{\left(Y_{i}-\bar{Y}\right)}^{2}}}$$ (20)

The variance inflation factor (VIF) determined for multicollinearity is the inverse of the correlation matrix and is calculated as follows.


$$VIF=\frac{1}{\left(1-R_{i}^{2}\right)}$$ (21)

The coefficient of determination, $R_{i}^{2}$, is determined by regressing *x*_*i*_ over the other *p* − 1 variables. The degree of multicollinearity increases with the value of VIF based on each variable. Generally speaking, VIF values higher than 10 indicate a weak model’s capacity for estimating and generalization [34].

The IBM SPSS Automated Linear Modeling (ALM) and Artificial Neural Network (ANN) analyses were conducted using the SPSS software program (version 25, SPSS Inc., Chicago, IL, USA). The MARS and Random Forest algorithms were implemented using the R Studio program [69].

## 3. Results

**Fig 2:**
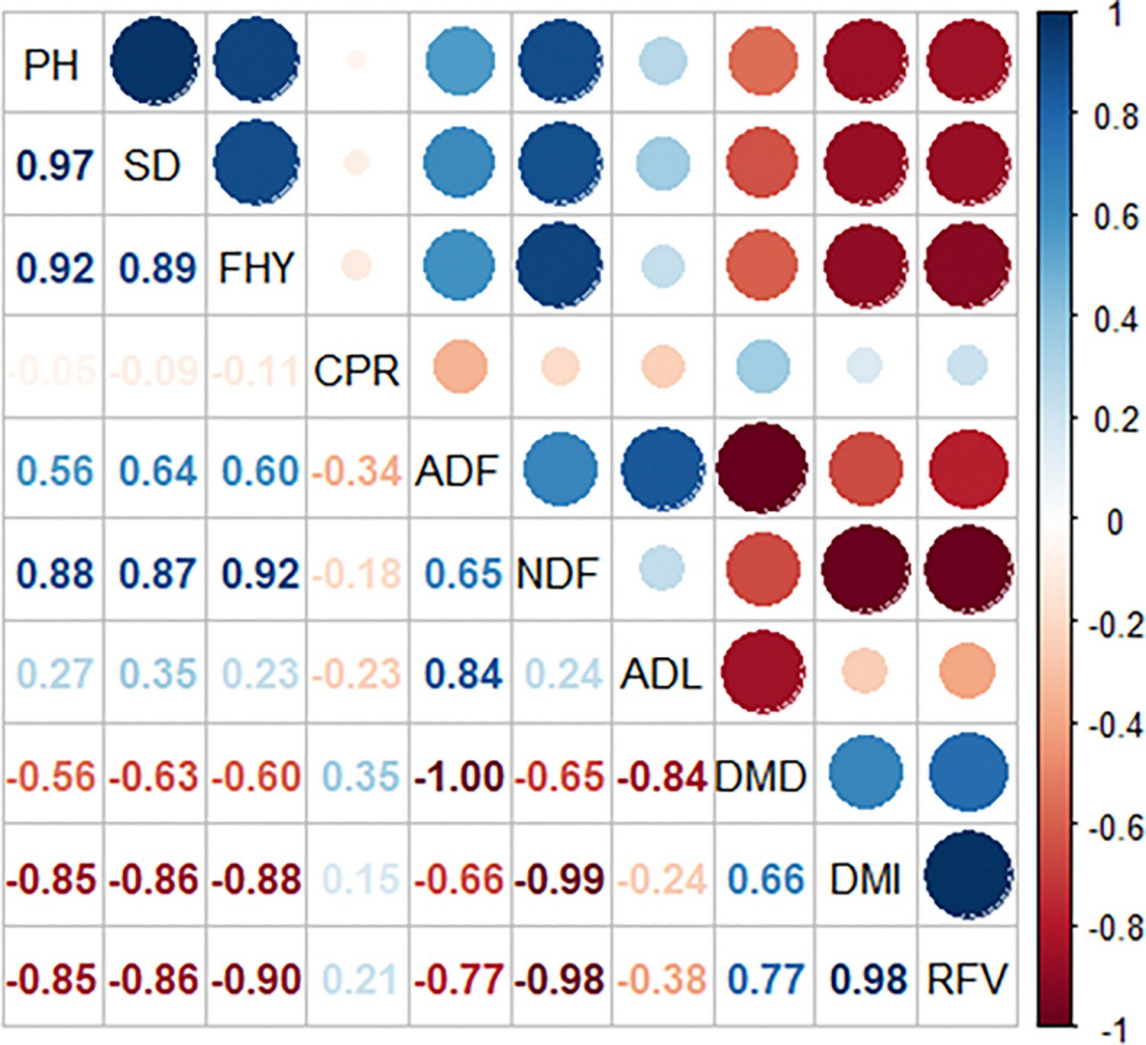
Correlation coefficients between plant characteristics.

Table 2 provides summary statistics for the plant characteristics of the SSG hybrid grown in Konya province, Türkiye, during the 2021 and 2022 growing seasons. The data showed normal distribution according to the Kolmogorov-Smirnov test and p>0.05. Also, the datasets are investigated for multicollinearity by using the diagnostic methods and results are given in Table 2. According to the results of fresh herbage yield data, it can be said that there is a problem of multicollinearity in the data due to the presence of variables (PH, SD, ADF, NDF, ADL, DMD and DMI) below the tolerance value of 0.1 and above the variance inflation factor (VIF) value of 10. The conclusions that there are strong correlations between the variables are further supported by the correlation analysis results shown in Fig 2.

**Table 2:** Summary statistics and multi-collinearity diagnostics for the plant characteristics of the SSG hybrid.

Variables	N	$\bar{X}$	$s_{\bar{x}}$	s	Minimum	Maximum	Tolerance	VIF
PH	32	251.220	11.277	63.795	150	332	0.023	44.010
SD	32	7.030	0.352	1.992	3.900	9.600	0.029	34.030
FHY	32	8010.690	345.300	1953.317	4800	10400		
CPR	32	9.090	0.091	0.515	8	10.100	0.662	1.510
ADF	32	33.230	0.383	2.171	29.600	36.600	0.000	2748.209
NDF	32	57.440	0.882	4.990	46.800	66.400	0.005	195.950
ADL	32	7.430	0.231	1.306	4.800	9.900	0.093	10.737
DMD	32	63.010	0.298	1.690	60.400	65.800	0.000	2678.503
DMI	32	2.110	0.034	0.197	1.800	2.600	0.014	71.544
RFV	32	103.020	2.058	11.646	86.200	129.500	0.003	286.414

$\bar{X}$: Mean, s: Standard deviation, $s_{\bar{x}}$: Standard error.

**Fig 3:**
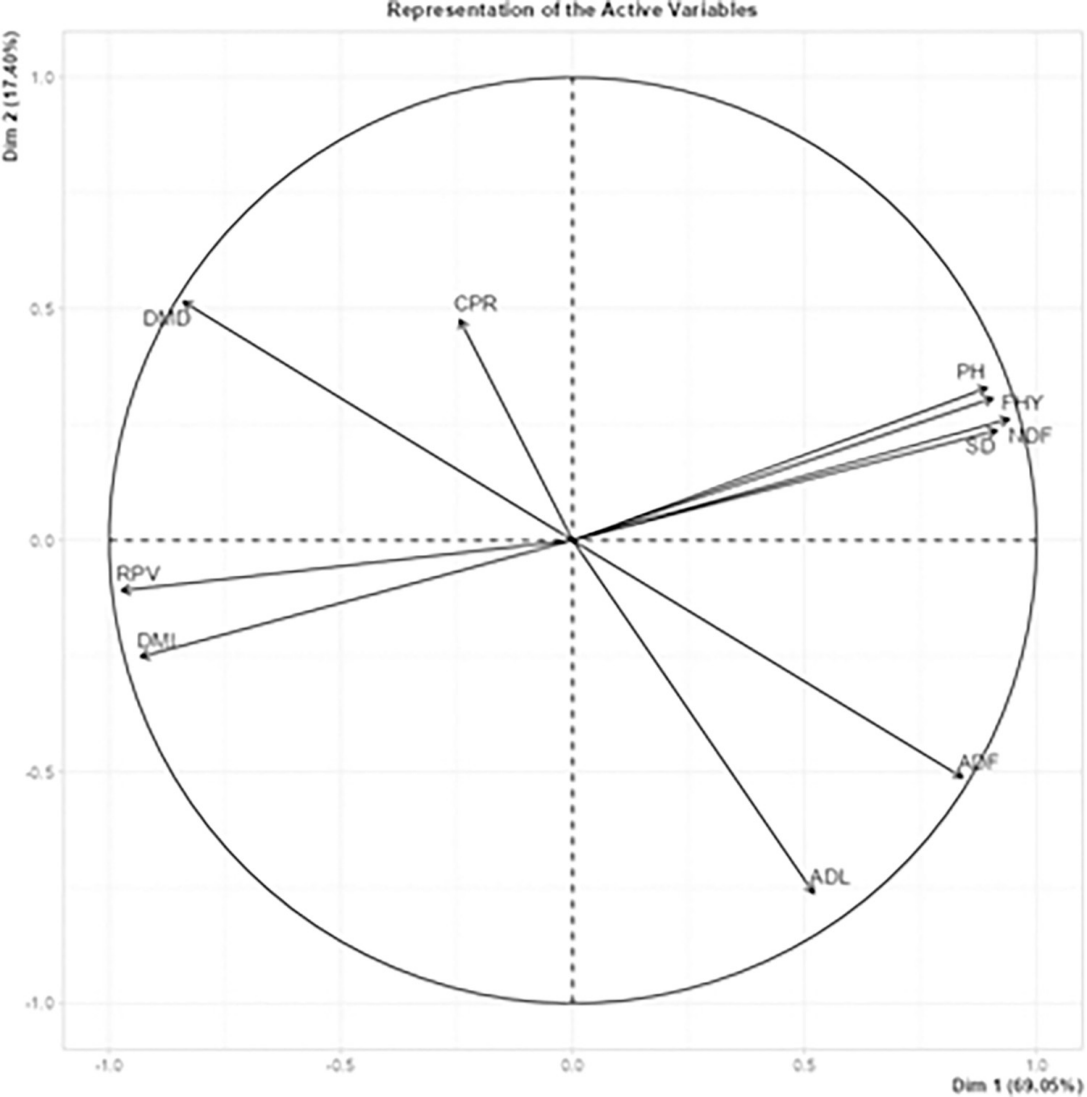
Results of Principal Component Analysis for plant characteristics.

Fig 2 presents the correlation coefficients for the plant traits of the SSG hybrid. Furthermore, Fig 3 shows the outcomes of the Principal Component Analysis (PCA) conducted on the plant characteristics of the SSG hybrid.

A separate test data set was used to evaluate the models’ generalization and prediction abilities after they were trained using the crossvalidation method due to the issue of multicollinearity. The results of the study show that the data mining (ANN, ALM, RF, and MARS) algorithms perform well for the data set with multicollinearity problem of statistical models. When multicollinearity is present, prior to using MARS, it was lowered the dimensionality of the input variables using principal components in order to enhance MARS’s capacity to handle multicollinearity. It was found that the resulting model enhances the accuracy of MARS in the multicollinear situation while maintaining interpretability using data on the features of sorghum plants. The results obtained are summarized as follows.

### 3.1. Result of Artificial Neural Network (ANN)

**Fig 4:**
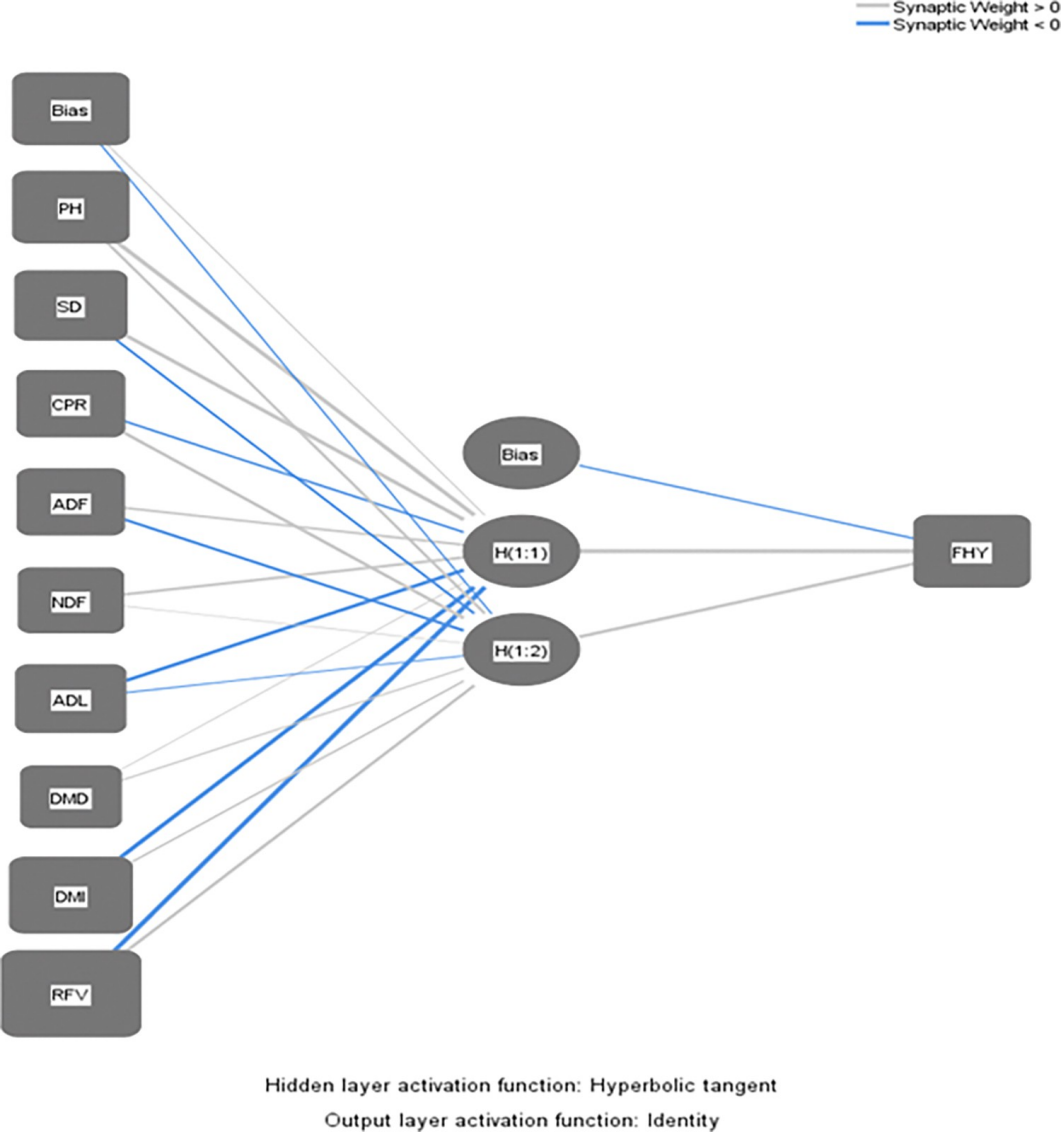
Structure of the artificial neural network.

The Multilayer Perceptron artificial neural network model was selected due to its suitability for the data. The optimization approach used was the scaled conjugate gradient, with 70% of the data allocated for training and 30% for testing the network. Fig 4 illustrates the connections within the ANN.

Fig 4 illustrates the artificial neural network design, where the identity function is used as the activation function in the output layer, and the hyperbolic tangent is used as the activation function in the hidden layer. Table 3 displays the parameter estimations for the ANN model.

**Table 3:** Parameter estimates of ANN.

Parameter Estimates			
Predictor		Predicted	
		Hidden Layer 1	Output Layer
		H(1:1)	FHY
Input Layer	(Bias)	-0.212	
	PH	-0.775	
	SD	0.194	
	CPR	0.333	
	ADF	0.188	
	NDF	-0.715	
	ADL	0.293	
	DMD	0.118	
	DMI	0.336	
	RPV	0.848	
Hidden Layer 1	(Bias)		-0.215
	H(1:1)		-1.041

Table 3 shows the connection weights between each neuron as follows:

The connection weight values between the input layer variables PH, SD, CPR, ADF, NDF, ADL, DMD, DMI, and RFV and H(1:1), the first neuron in the hidden layer, are -0.775, 0.194, 0.333, 0.188, -0.715, 0.293, 0.118, 0.336, and 0.848, respectively.

In the ANN model, the learning Sum of Squares Error (SSE) value was 0.783, and the relative error was 0.065. The test’s SSE was 0.051, with a relative error of 0.019. The percentage relevance of the independent variables is displayed in Table 4.

**Table 4:** Significance of independent variables.

	Importance	Normalized Importance
PH	0.198	69.6%
SD	0.025	8.9%
CPR	0.077	27.3%
ADF	0.024	8.5%
NDF	0.239	84.2%
ADL	0.039	13.8%
DMD	0.022	7.6%
DMI	0.091	32.2%
RFV	0.284	100.0%

**Fig 5:**
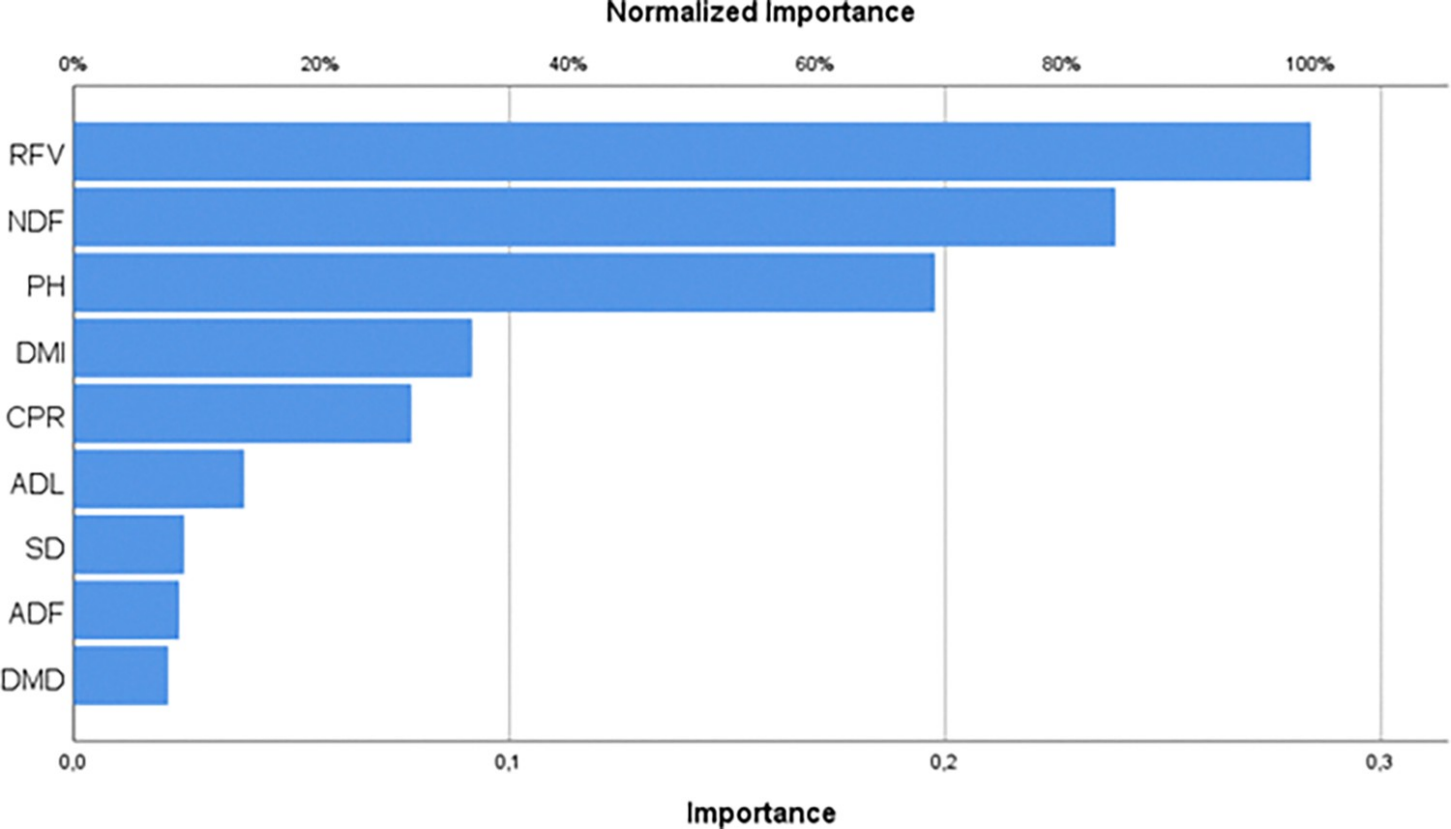
Importance of variables.

Table 4 shows that the impact values of the independent factors on fresh herbage yield (FHY) in the output layer are as follows: RFV is 0.284, NDF is 0.239, PH is 0.198, DMI is 0.091, CPR is 0.077, ADL is 0.039, SD is 0.025, ADF is 0.024, and DMD is 0.022. Fig 5 displays a percentage column graph illustrating the impact of these factors on the prediction.

Fig 5 illustrates that in this model, RFV has the largest impact on the fresh herbage yield (FHY) of SSG hybrid, with a 100% influence from the terminal. Additionally, NDF is the second most significant independent variable, with an impact rate of 84.2%, while PH has an effect of 69.6%. DMD had the least impact on fresh herbage production from the terminals, with a rate of 7.6%. Other variables, including DMI (32.2%), CPR (27.3%), ADL (13.8%), SD (8.9%), and ADF, have an effect of 8.5%.

### 3.2. Automatic Linear Modeling (ALM) results

Table 5 shows the results of the model prediction coefficients, and the significance achieved when ALM was used.

**Table 5:** Coefficients determined for the fresh herbage production (FHY) target variable.

Model Term	Coefficient	P-value	Importance
Intercept	12222.458	0.001	
RFV	-81.384	0.001	0.524
PH	29.128	0.001	0.392
SD	-447.716	0.103	0.084

^a^ This coefficient was set to zero because it was redundant.

**Fig 6:**
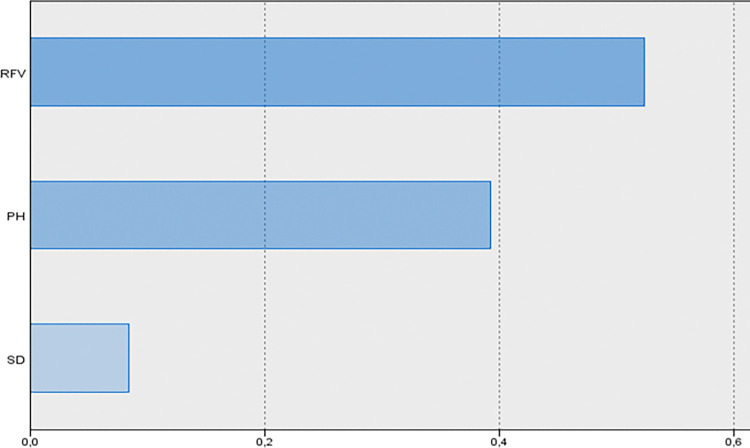
Predictor importance in the estimating model for FHY.

The ALM method was used to assess the predictability of the mean FHY, with the key contributing factors summarized in Table 5. Notably, the SD variable was not statistically significant in the ALM analysis. Table 5 also provides parameter estimates for the overall model, showing the individual impact of each factor on the target variable. The coefficients illustrate the relationship between each predictor and the mean fresh herbage yield, assuming the other variables remain constant. The importance of each predictor, as identified by the ALM method, is also highlighted in Table 5, with standardized values summing to one. The model reached an accuracy of 89.5%, calculated by multiplying the adjusted R^2^ by 100. The predictor importance graph (Fig 6) further demonstrates the relative significance of each factor, with RFV (0.524), PH (0.392), and SD (0.084) emerging as the most influential, with RFV being the key predictor of FHY.

**Fig 7:**
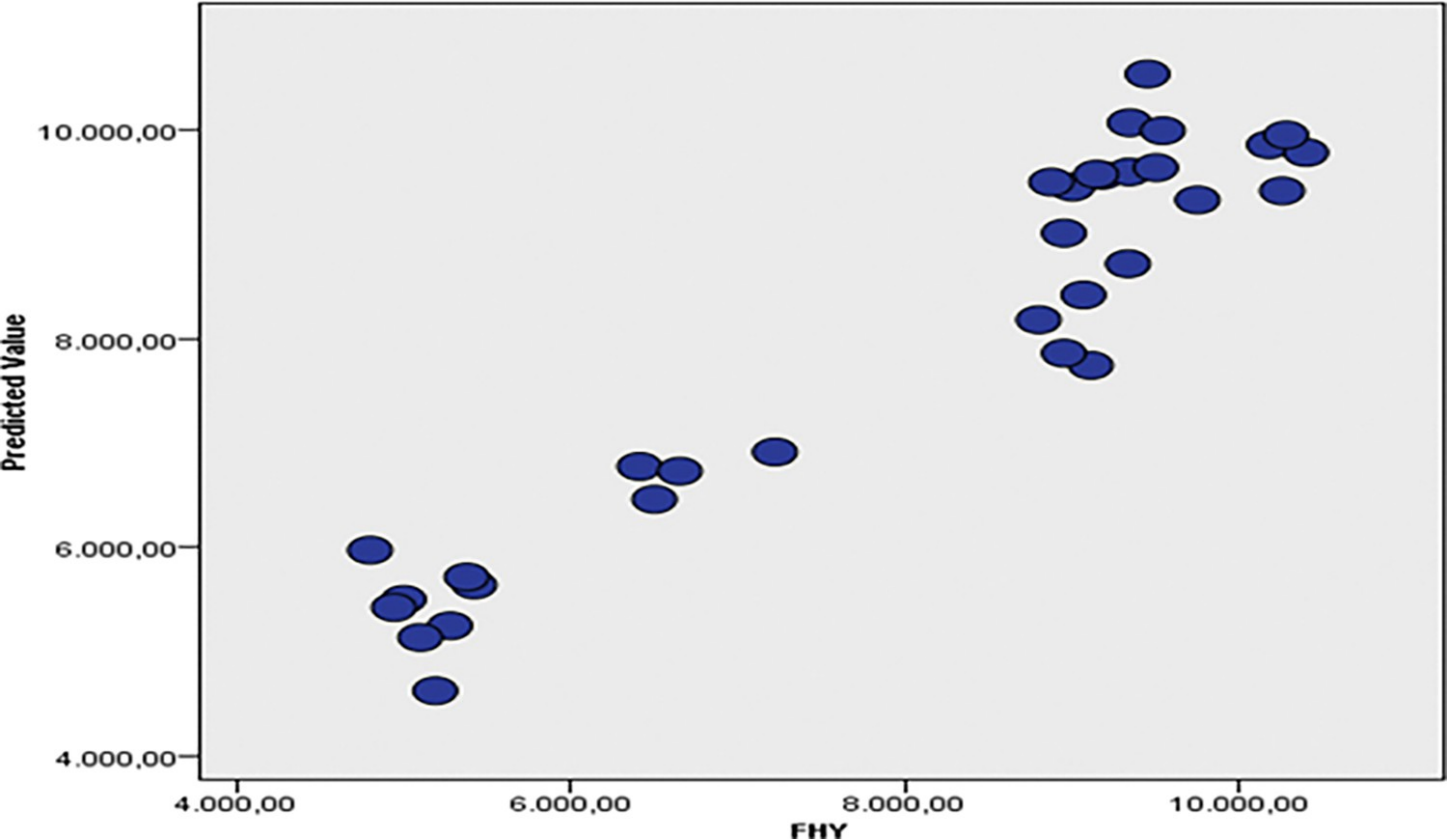
Excluded scatterplot of observed versus predicted values for FHY.

**Fig 8:**
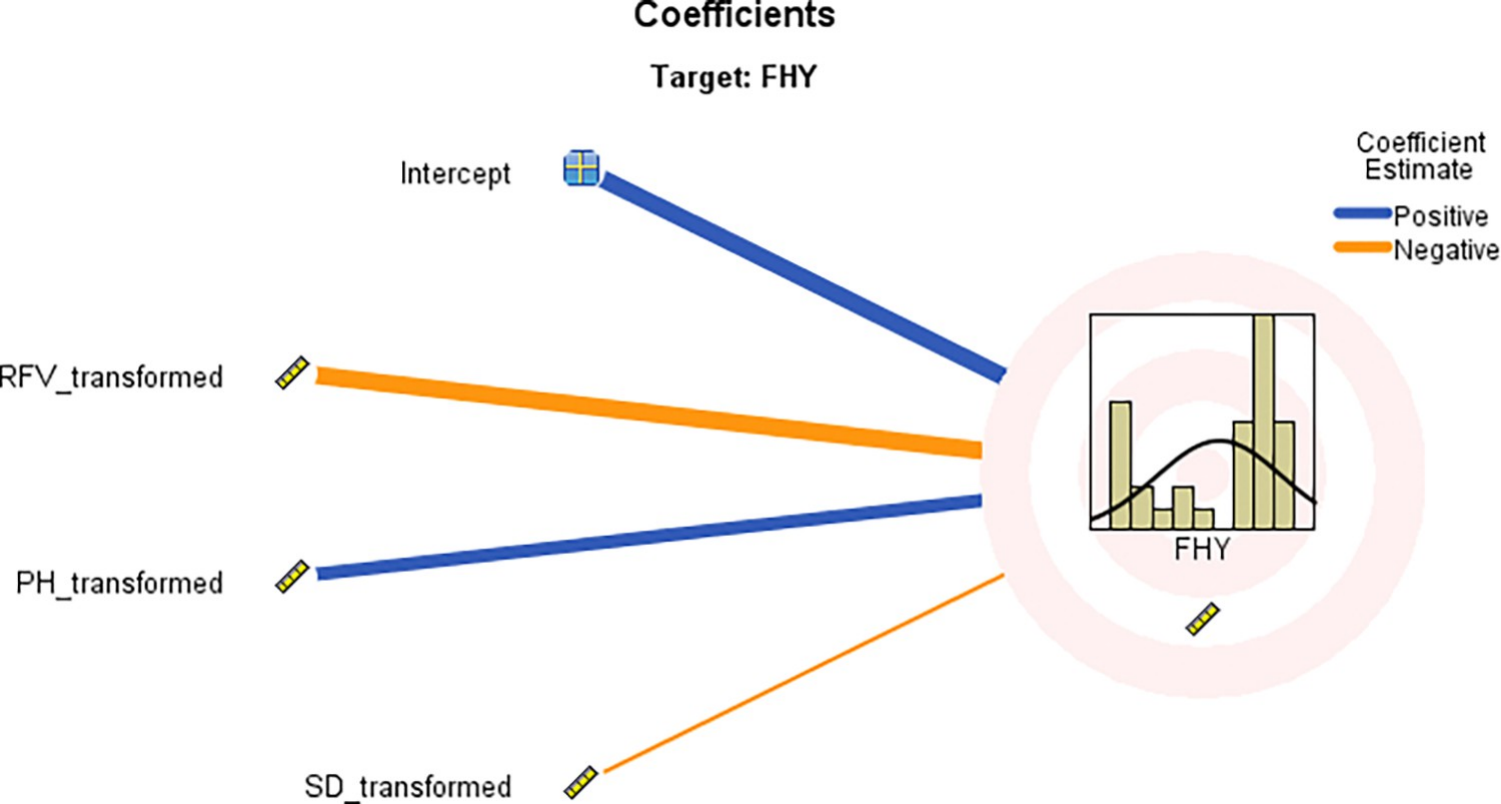
Coefficient values for FHY.

The discarded scatter plot of FHY displays predictor values on the y-axis and observed values on the x-axis, indicating that a larger percentage of sample locations are on the 45-degree line, suggesting that the model is relatively accurate (Fig 7). Fig 8 shows that FHY has a positive association with PH and SD, but a negative correlation with RFV.

### 3.3. Random Forest (RF) algorithm results

**Fig 9:**
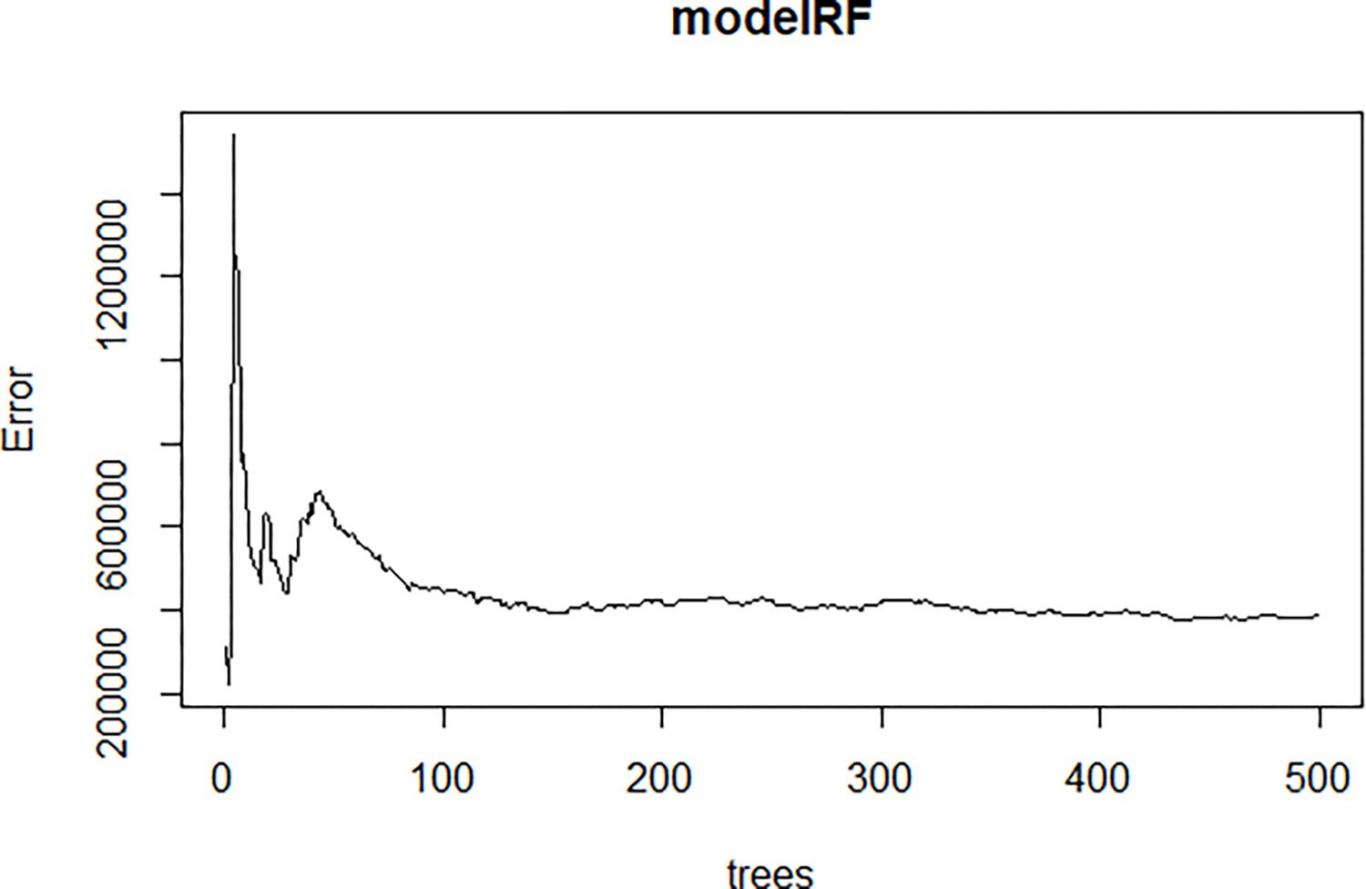
RF Algorithm error rate of the model.

The findings of the RF algorithm are summarized here. Fig 9 illustrates the random forest trees designed to minimize the error value.

**Fig 10:**
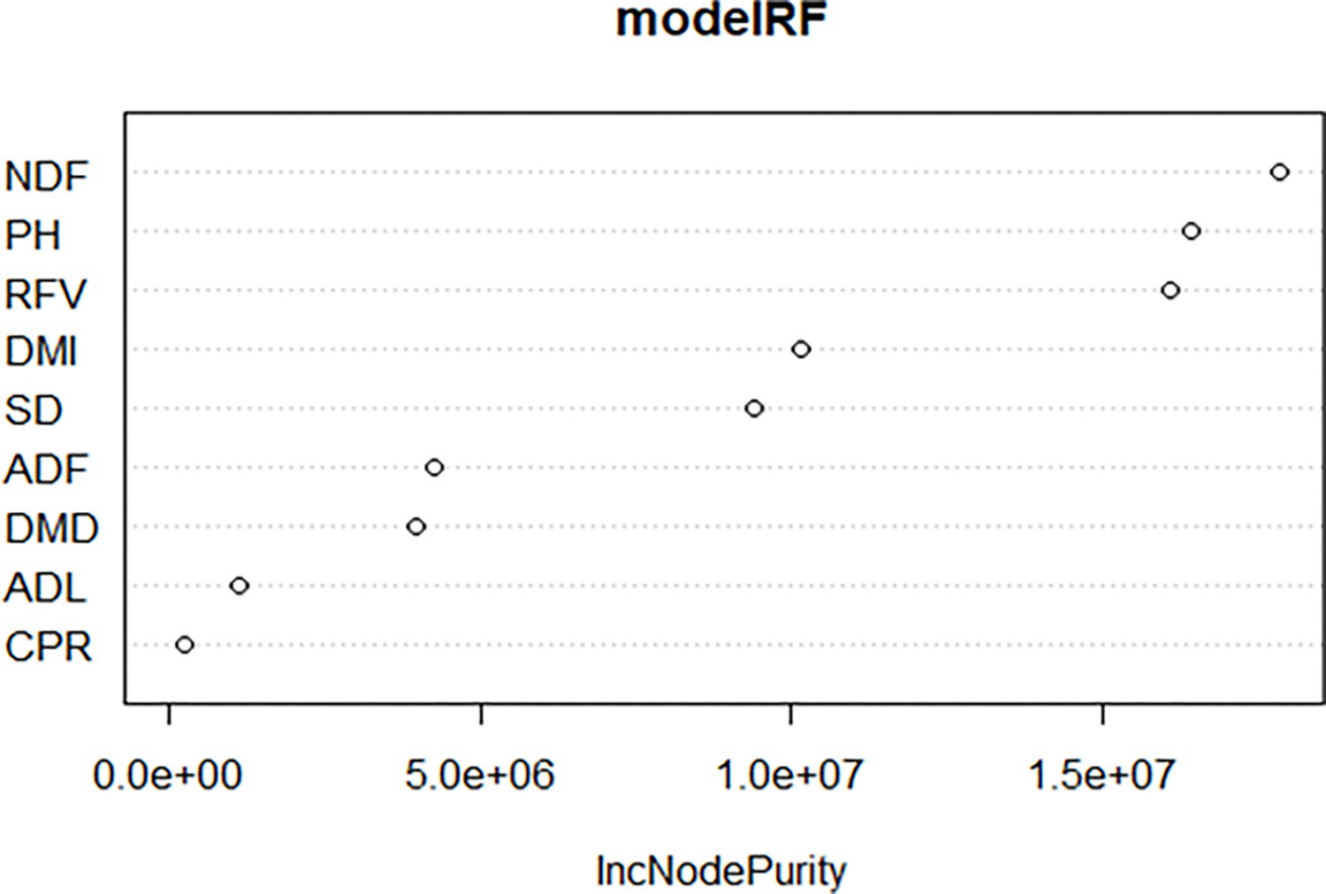
Significance graph of the variables of the RF model.

The RF method was employed to build the model, using fresh herbage yield (FHY) as the dependent variable. The RF model incorporates various plant linear characteristics as predictors, including PH, SD, CPR, ADF, NDF, ADL, DMD, DMI, and RFV. The random forest algorithm used 500 trees. The model explains 88.87% of the variation in the dependent variable, with MSE = 160,067, RMSE = 400, MAE = 321, and Bias = 170. Neutral detergent fiber (NDF) is the most significant factor influencing the model, followed by PH and RFV (as shown in Table 6 and Fig 10).

**Table 6:** Predictor importance in RF.

Variable	Node Purity
PH	16403737.2
SD	9377856.4
CPR	237485.3
ADF	4259936.6
NDF	17835601.8
ADL	1124657.1
DMD	3982120.2
DMI	10135817.0
RFV	16091141.2

It is also possible to estimate fresh herbage production by varying the values of the characteristic features that represent the independent variables in the equation generated by the Random Forest (RF) technique. For example, consider the following calculation: PH = 225, SD = 7, CPR = 8.5, ADF = 31, NDF = 55, ADL = 8.5, DMD = 66, DMI = 2.1, RFV = 111, resulting in FHY = 7539.289 kg.

### 3.4. MARS algorithm results

Table 7 presents the model estimation coefficients of the MARS approach used for predicting fresh herbage yield (FHY).

**Table 7:** Outcomes of the MARS algorithm in predicting the fresh herbage yield (FHY) of the SSG hybrid.

Basis functions	Estimate	Std. Error	t value	Pr(>|t|)
Intercept	9212.80	430.52	21.399	2.96e-15***
bx[, –3]h(PH-231)	189.82	55.02	3.450	0.00253**
bx[, –3]h(RPV-97)	-1137.12	207.15	-5.489	2.26e-05***
bx[, –3]h(97-RPV)	1898.69	758.70	2.503	0.02112*
bx[, –3]h(56.5-NDF)	-13867.50	2854.80	-4.858	9.54e-05***
bx[, –3]h(231-PH)*DMI	-11.41	1.95	-5.851	1.01e-05***
bx[, –3]h(NDF-56.5)*ADL	-56.97	21.04	-2.708	0.01353*
bx[, –3]h(56.5-NDF)*DMD	225.05	44.85	5.018	6.60e-05***
bx[, –3]h(SD-8.2)	-511.13	192.60	-2.654	0.01524*
bx[, –3]h(RPV-95.3)	790.34	177.89	4.443	0.00025***
bx[, –3]SD*h(97-RPV)	-187.81	77.86	-2.412	0.02558*
bx[, –3]h(PH-231)*DMI	-84.89	27.37	-3.102	0.00562**

Signif. codes: 0

‘***’ 0.001

‘**’ 0.01

‘*’ 0.05 ‘.’ 0.1 ‘ ‘ 1

Below is a detailed equation that was developed by considering the interaction effects of the model’s coefficients.

FHY = 9380–234 * max(0, 231—PH) + 194 * max(0, PH—231)

- 504 * max(0, SD—8.2) - 7613 * max(0, 56.5—NDF) + 773 * max(0, RPV—95.3)

+ 1776 * max(0, 97—RPV) - 1072 * max(0, RPV—97) + 91.7 * max(0, 231—PH)* DMI

- 87.9 * max(0, PH—231) * DMI—174 * SD * max(0, 97—RPV)

- 58.8 * max(0, NDF—56.5) * ADL + 123 * max(0, 56.5—NDF) * DMD

By adjusting the values of the characteristic features that represent the independent variables in the equation produced by the MARS algorithm, it is possible to estimate the fresh herbage yield. For instance, consider the following calculation: PH = 225, SD = 7, CPR = 8.5, ADF = 31, NDF = 55, ADL = 8.5, DMD = 66, DMI = 2.1, RFV = 111, resulting in FHY = 7053.170 kg.

**Fig 11:**
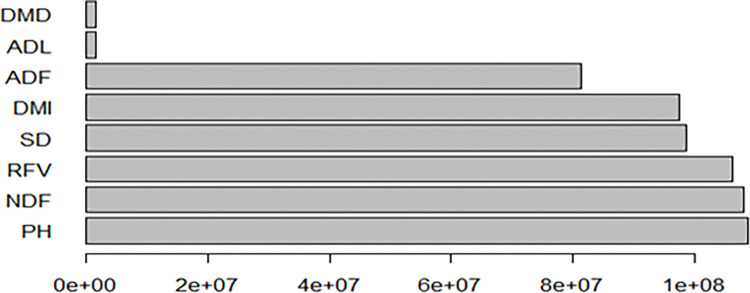
Graph of relative importance.

Fig 11 illustrates the proportional relevance of the factors used in the MARS algorithm to forecast fresh herbage yield.

**Fig 12:**
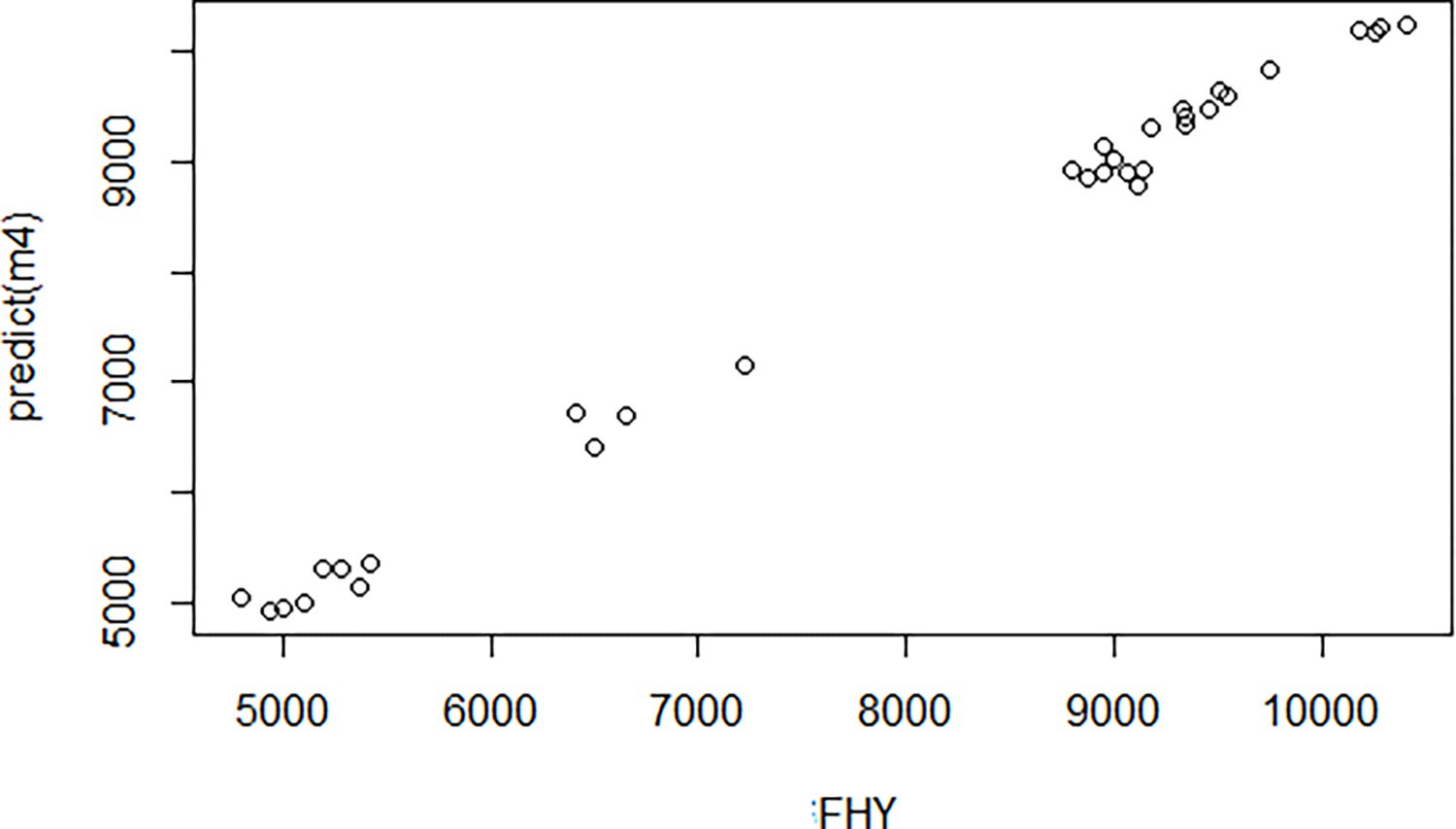
The agreement between the predicted and actual fresh herbage yield (FHY) values.

Fig 12 presents a graph comparing the observed values with the estimated values generated by the MARS algorithm.

**Fig 13:**
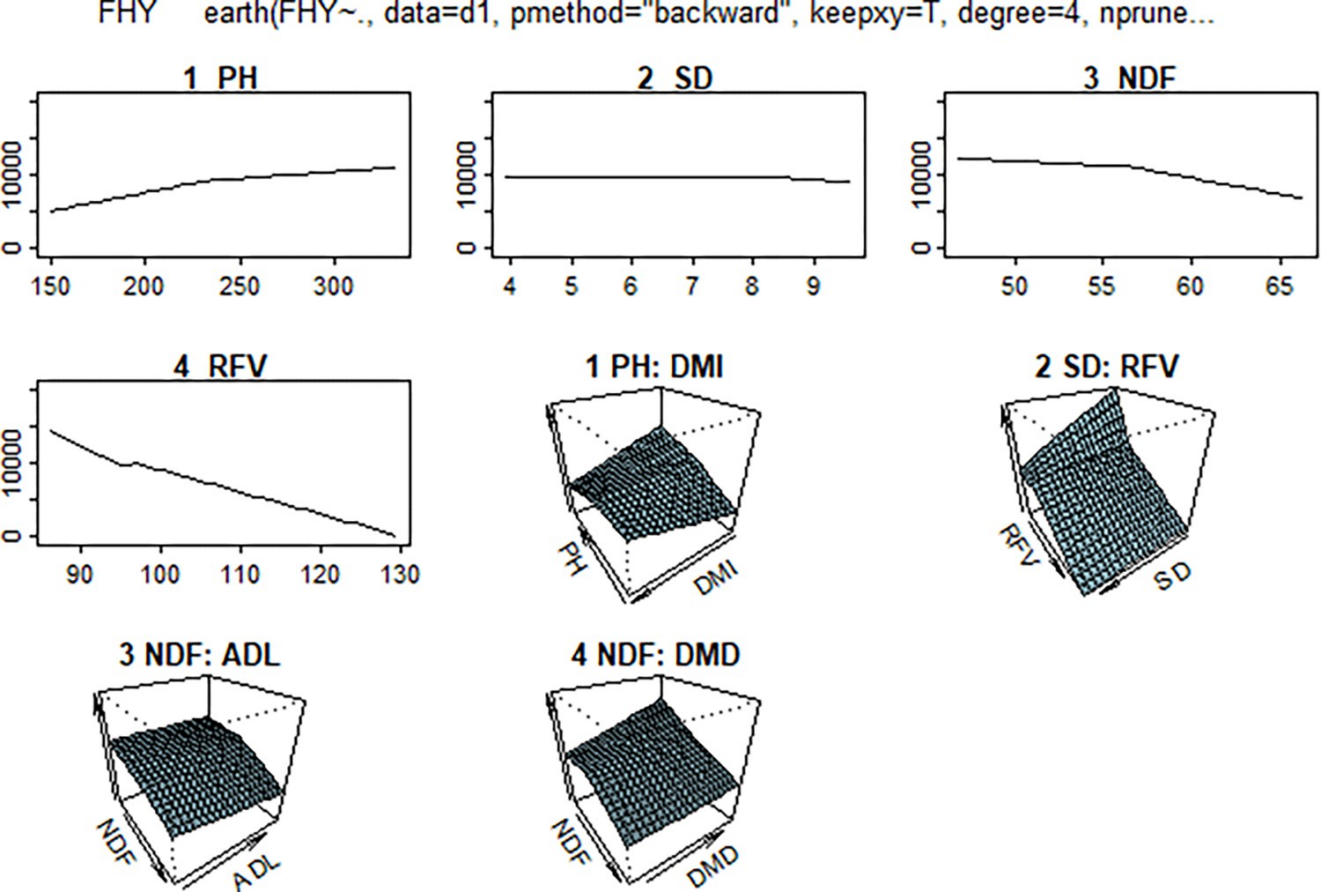
Model surface plots in MARS algorithm.

The study found a bilateral interaction between the variables. Fig 13 illustrates the three-dimensional surface graph of the analysis findings, highlighting the connection between two predictor variables and the objective variable.

When the goodness of fit statistics of the MARS algorithm were evaluated, it was calculated as R2 = 0.995, Adj. R2 = 0.991, MSE = 18961, RMSE = 137.7, MAPE = 1.488, MAE = 109.718, RAE = 0.017 and SD Ratio = 0.072.

The goodness-of-fit criterion results for all methods are displayed in Table 8. Each algorithm produced quite accurate FHY forecasts. In terms of expected accuracy, MARS was found to be the best method, followed by RF, ANN, and then ALM.

**Table 8:** Predictive performance of MARS, RF, ANN and ALM types.

Statistics	RF	ANN	MARS	ALM	Acceptable range	The most suitable method
r	0.960	0.972	0.997	0.951	The highest value	MARS
R^2^	0.921	0.945	0.995	0.904	The highest value	MARS
Adj. R^2^	0.913	0.936	0.991	0.901	The highest value	MARS
MSE	160067	209085	18961	350571	The lowest value	MARS
RMSE	400	457.259	137.700	592.091	The lowest value	MARS
MAPE	2.865	5.038	1.488	6.280	The lowest value	MARS
MAE	321	360.665	109.718	487.923	The lowest value	MARS
RAE	0.164	0.204	0.017	0.276	The lowest value	MARS

## 4. Discussion

In the study conducted by [70], plant height exhibited a significant positive correlation with total dry matter yield, SD, CPY, CPC, DMD, and ME, while showing a significant negative correlation with panicle length, number of tillers, and ADF. Stem diameter also showed a strong positive correlation with plant height, crude protein yield, panicle length, ether extract, and total dry matter yield. [16] explored the use of the Random Forest (RF) data mining technique to analyze the relationship between various climate factors and maize yield, finding an average R-squared of 28% and an explained R-squared of 55%. Similarly, [26] applied the RF algorithm to a dataset from 1980 to 2016, creating a sorghum yield prediction model with an R-squared value of 0.71. In another study by [25], several algorithms, including Support Vector Regression, RF, Extreme Learning Machine, ANN, and DNN, were tested for wheat yield prediction in two provinces. The Deep Neural Network (DNN) performed best in the first province with an RMSE of 0.04 q/ha and an R-squared of 0.96, while RF outperformed other models in the second province with an RMSE of 0.05 q/ha. [27] applied data mining techniques like BG, DT, RF, and ANN to predict maize yield using variables such as annual average temperature, precipitation, rainy days, frosty days, and hot days. Their study found that the ANN algorithm achieved the highest accuracy (r = 0.98, relative absolute error = 21.87%, root relative squared error = 20.44%, and RMSE = 423.23), highlighting ANN’s effectiveness in yield prediction. Similarly, [71] identified temperature and precipitation as key factors affecting maize yield using comparable models. [72] also applied Random Forest regression and found that maximum temperature and precipitation were critical climate factors influencing maize yield.

The parameters influencing the fresh grass production of pea plants grown in Turkey were investigated using multivariate adaptive regression spline (MARS), Chi-square automatic interaction detection (CHAID), classification and regression tree (CART), and artificial neural network (ANN) models. The MARS approach was shown to be the most effective model for measuring plant fresh herbage yield, with the highest R^2^ and adjusted R^2^ values (0.998 and 0.986) and the lowest values of RMSE, MAPE, SD ratio, AIC, and AICc (10.499, 0.7365, 0.047, 268, and 688, respectively) [73].

The CHAID, CART, MARS, and Bagging MARS algorithms were used in the study by [74] to analyze the parameters impacting fresh herbage yield in sorghum-sudangrass hybrids. The best algorithms for predicting the dependent variable were determined to be MARS, Bagging MARS, CART, and CHAID, in that order. The MARS algorithm was shown to be the most accurate predictor of crop yield.

Another study employed multiple regression analysis (MLR), artificial neural networks (ANNs), and the Multivariate Adaptive Regression Splines (MARS) method to estimate the stem weight of alfalfa plants. In the estimation of stem weight, the ANN, MARS, and MLR correlation coefficients (r) were 0.801, 0.999, and 0.753 for the Gea clover variety, and 0.781, 0.998, and 0561 for the Basbag variety. The Gea variety’s R^2^ in the same models was 0.642, 0.998, and 0.567, while the Basbag variety’s was 0.610, 0.997, and 0.315. The Gea variety’s MSE values were 0.023, 0.008, and 2.498, whereas the Basbag variety’s were 0.151, 0.017, and 4.641. Compared to ANNs and MLR, the MARS algorithm produced a more accurate forecast. MARS > ANN > MLR was the sequence of algorithms utilized to improve prediction results for alfalfa plant stem weight estimate [75].

[76] used donkey biometric data to examine the predicted performance of many machine learning algorithms, including CHAID, Random Forest, ALM, MARS, and Bagging MARS. With the lowest RMSE, MAPE, and SD ratio values (2.173, 1.615, and 0.291, respectively) and the greatest R2 value (0.916), the MARS algorithm was determined to be the most effective model for determining donkey body length. From best to worst, the algorithms’ performance results were as follows: MARS > Bagging MARS > Random Forest > CHAID > ALM.

To predict egg weight from certain egg quality criteria in chickens, the following methods were employed: random forest (RF), multivariate adaptive regression spline (MARS), categorization and regression trees (CART), bagging MARS, chi-square automated interaction detector (CHAID), and exhaustive CHAID. The values of the correlation coefficient (r) varied from 0.99999 (MARS and Bagging MARS) to 0.957 (CHAID). MARS and bagging MARS algorithms had the lowest RMSE (0.001), whereas CHAID had the most (2.154). MARS ≈ Bagging MARS > RF > CART > Exhaustive CHAID > CHAID was determined to be the algorithm’s supremacy order in terms of prediction accuracy [77].

The results of comparative data mining algorithms used in different plants and livestock data are consistent with the results of this study in terms of the suitability of the methods and especially the MARS algorithm giving the best results.

## 5. Conclusion

This study assessed the performance of the ANN, ALM, RF, and MARS methods in predicting the FHY of the SSG hybrid. The key findings of the study are as follows:

In the SSG hybrid, seven factors (PH, SD, ADF, NDF, DMD, DMI, and RFV) significantly influence fresh herbage yield. Among these, the most impactful factors are PH, RFV, and NDF.

The RF method accurately predicts the FHY of the SSG hybrid, accounting for 89.50% of the variation. In comparison, the ALM method achieved an accuracy of 88.87%, slightly lower than the RF method.

The factors that determine the fresh herbage yield in SSG hybrid are ranked in order of significance as follows: RFV, PH, and SD for the ALM technique; RFV, NDF, and PH for the ANN method; PH, NDF, and RFV for the MARS algorithm; and NDF, PH, and RFV for the RF algorithm. When analyzing the importance ranking of plant traits affecting fresh herbage yield in SSG hybrid, the RF, ANN, and MARS methods showed similar characteristics in terms of variable importance ranking. The only difference was the order of the top three variables (RFV, NDF, and PH). In the ALM method, however, the SD variable was included, whereas NDF was not among the top three.

The performance results, from worst to best, are as follows: ALM < ANN < RF < MARS.

The results of the study show that statistical models, especially the MARS algorithm, perform better among data mining algorithms for the data set with multicollinearity problem. As a result, selecting the right approach is crucial because multicollinearity is a common issue in practical applications. Thus, it can be said that data mining techniques and statistical models are strong instruments that provide efficient answers to the multicollinearity issue.

It has been shown that data mining methods are extremely effective in predicting variables and uncovering the relationships between plant traits and characteristics in agricultural field data.
